# Expression of miR-138 in cryopreserved bovine sperm is related to their fertility potential

**DOI:** 10.1186/s40104-023-00909-1

**Published:** 2023-09-20

**Authors:** Albert Salas-Huetos, Jordi Ribas-Maynou, Yentel Mateo-Otero, Carolina Tamargo, Marc Llavanera, Marc Yeste

**Affiliations:** 1 Unit of Cell Biology, Department of Biology, Faculty of Sciences, University of Girona, Girona, 17003 Spain; 2 Biotechnology of Animal and Human Reproduction (TechnoSperm), Institute of Food and Agricultural Technology, University of Girona, Girona, 17003 Spain; 3 Department of Nutrition, Harvard T.H. Chan School of Public Health, Harvard University, Boston, MA 02115 USA; 4 Consorcio CIBER, M.P., Fisiopatología de la Obesidad y Nutrición (CIBERobn), Instituto de Salud Carlos III (ISCIII), Madrid, 28029 Spain; 5 Present Address: Unit of Preventive Medicine and Public Health, Faculty of Medicine and Health Sciences, Universitat Rovira i Virgili, Reus, 43201 Spain; 6 Department of Animal Selection and Reproduction, The Regional Agri-Food Research and Development Service of Asturias (SERIDA), Gijón, 33394 Spain; 7 Catalan Institution for Research and Advanced Studies (ICREA), Barcelona, 08010 Spain

**Keywords:** Bovine, Fertility, miRNAs, Sperm

## Abstract

**Background:**

MicroRNAs (miRNAs) are small, single-stranded, non-coding RNA molecules of 22–24 nucleotides that regulate gene expression. In the last decade, miRNAs have been described in sperm of several mammals, including cattle. It is known that miRNAs can act as key gene regulators of early embryogenesis in mice and humans; however, little is known about the content, expression, and function of sperm-borne miRNAs in early bovine embryo. In this study, total sperm RNA was isolated from 29 cryopreserved sperm samples (each coming from a separate bull) using a RNeasy kit and treatment with DNase I. RNA concentration and purity were determined through an Epoch spectrophotometer and an Agilent Bioanalyzer. The expression of 10 candidate miRNAs in bovine sperm (bta-miR-10a, bta-miR-10b, bta-miR-138, bta-miR-146b, bta-miR-19b, bta-miR-26a, bta-miR-34a, bta-miR-449a, bta-miR-495 and bta-miR-7), previously identified in testis and/or epididymis, was evaluated with RT-qPCR. The cel-miR-39-3p was used as a spike-in exogenous control. Nonparametric Mann–Whitney tests were run to evaluate which miRNAs were differentially expressed between bulls with high fertility [HF; non-return rates (NRR) ranging from 39.5 to 43.5] and those with subfertility (SF; NRR ranging from 33.3 to 39.3). Several sperm functionality parameters (e.g., viability, membrane stability or oxygen consumption, among others) were measured by multiplexing flow cytometry and oxygen sensing technologies.

**Results:**

RNA concentration and purity (260/280 nm ratio) (mean ± SD) from the 29 samples were 99.3 ± 84.6 ng/µL and 1.97 ± 0.72, respectively. Bioanalyzer results confirmed the lack of RNA from somatic cells. In terms of the presence or absence of miRNAs, and after applying the Livak method, 8 out of 10 miRNAs (bta-miR-10b, -138, -146b, -19b, -26a, -449a, -495, -7) were consistently detected in bovine sperm, whereas the other two (bta-miR-10a, and -34a) were absent. Interestingly, the relative expression of one miRNA (bta-miR-138) in sperm was significantly lower in the SF than in the HF group (*P* = 0.038). In addition to being associated to fertility potential, the presence of this miRNA was found to be negatively correlated with sperm oxygen consumption. The expression of three other miRNAs (bta-miR-19b, bta-miR-26a and bta-miR-7) was also correlated with sperm function variables.

**Conclusions:**

In conclusion, although functional validation studies are required to confirm these results, this study suggests that sperm bta-miR-138 is involved in fertilization events and beyond, and supports its use as a fertility biomarker in cattle.

**Supplementary Information:**

The online version contains supplementary material available at 10.1186/s40104-023-00909-1.

## Background

In cattle and other livestock, the methods traditionally used for determining sperm quality (macroscopic and microscopy-based methods) have proven not to be enough to predict the fertilizing potential [1]. Interestingly, most of the artificial inseminations in cattle are performed using cryopreserved semen, which has lower sperm quality and reduced fertility compared with those inseminations with fresh semen. The accurate assessment of sperm fertility potential is, therefore, critical to increase the artificial insemination success. Recent advances in -omics (e.g., genomics and epigenomics, transcriptomics, metabolomics, and proteomics) have uncovered relevant biomarkers in semen [2] and have revealed the potential of microRNAs (miRNAs) as fertility predictors in both humans [2] and cattle [3].

MiRNAs are small, single-stranded, non-coding RNA molecules of 22–24 nucleotides that regulate gene expression. In the last decade, miRNAs have been identified in the sperm of several mammals, including humans [4], mice [5] and cattle [6, 7]. Thus far, up to 1,085 miRNAs have been described in bovine species (*Bos taurus*) (Sanger miRBase v.22.1; www.mirbase.org, last accession: November 2022). Moreover, miRNAs are understood to play a critical role in cell cycle control [8], differentiation [9], spermatogenesis and oogenesis [10–13], and early embryogenesis [13–15] in mice and humans. New evidence suggests that sperm-derived miRNAs are not delivered to the oocyte randomly as remnants of spermatogenesis [16], which supports that not only do they play a role in spermatogenesis but also during embryo development. For example, miR-34c, one of the most abundant miRNAs in human sperm [4], can modulate fertilization in mice regulating the first cleavage [4, 17]. Interestingly, an exclusive paternal inheritance of the aforementioned miRNA is described in mice pointing out the importance of sperm-borne miRNAs for fertility outcomes [13, 18]. Although some authors previously suggested that the miR-34 family members may be essential for the development of bovine gametes [19], however, little is known about the content, expression, and function of other sperm-borne miRNAs in early bovine embryo and its relationship with fertility.

In this study, the expression of miRNAs in sperm has been hypothesized to be related to sperm fertility in cattle. The main objective of this work, therefore, is to analyze if some candidate miRNAs are associated with bull fertility.

## Methods

### Animals, ejaculates and sample description

The analysis included 29 semen samples from healthy adult (1.5- to 2-year-old) Holstein bulls that were phenotypically characterized previously. Animals were housed at Cenero AI center in Gijón, Asturias (Spain), in compliance with European Union regulations for animal husbandry. Bulls were fed and housed under standard conditions to produce commercially available cryopreserved sperm straws.

Extensive information over several fertility parameters which included in vivo fertility measured as non-return rates (NRR; the proportion of cows inseminated for the first time that did not return to estrus within 90 d post-artificial insemination) were recorded for all bulls. NRRs were calculated through dividing the number of pregnant cows by the total number of inseminations. An average of 2,293 females per bull was inseminated (minimum of 277 and maximum of 15,231 females per bull). Sperm samples were pooled using three 0.25-mL straws from three different frozen semen ejaculates from the same bull collected at 5-week intervals throughout the year, and contained, on average, 5 mL of volume with over 10^9^ spermatozoa/mL and more than 85% of total motile sperm. Depending on the NRR median value (NRR range = 33.33 to 43.48), 2 groups were established and subsequently compared in the analyses described below: subfertile (SF; *n* = 15 cases) and highly fertile (HF; *n* = 14 controls).

Frozen ejaculates were prepared by adjusting the concentration to 92 × 10^6^ sperm/mL in a commercial extender (Bioxcell, IMV Technologies; L'Aigle, France) and then packaged into 0.25-mL straws. Cryopreservation was performed by using a controlled-rate freezer (Digit-cool, IMV Technologies; L'Aigle, France) after which straws containing sperm samples were stored in a nitrogen tank. Briefly, the freezing program used was: from 4 to −10 ºC at −5 ºC/min; from −10 to −100 ºC at −40 ºC/min; and from −100 to −140 ºC at −20 ºC/min. Following thawing at 38 ºC for 20 s, sperm quality and functionality was assessed for each sample. Sperm parameters were measured immediately post-thawing. All reagents used in this work were purchased from Sigma-Aldrich (Saint-Louis, MO, USA), unless otherwise stated.

### Evaluation of sperm motility and morphology

Sperm samples were first prewarmed for 20 s at 38 °C and diluted 1:3 (v:v) with PBS. With 3 µL of each sperm sample, motility and kinematic parameters were measured using 20-µm Leja chamber slides (Leja Products BV; Nieuw-Vennep, The Netherlands) and a Computer-Assisted Sperm Analysis (CASA) system (Integrated Sperm Analysis System V1.0; Spain) under a negative phase-contrast field microscope (Olympus BX41; Japan). Two replicates were examined analyzing a minimum of 1,000 sperm cells per replicate. Percentages of total and progressive motility were determined for each sample. Besides, several kinematics parameters were recorded: 1) average path velocity (VAP, µm/s), 2) curvilinear velocity (VCL, μm/s), 3) straight-line velocity (VSL, μm/s), 4) linearity (LIN = VSL/VCL × 100, %), 5) straightness (STR = VSL/VAP × 100, %), 6) oscillation (WOB = VAP/VCL × 100, %), 7) lateral head displacement (ALH, μm), and 8) frequency of head displacement (BCF, Hz). A spermatozoon was considered to be motile when the VAP was higher than 10 μm/s, and was considered to be progressively motile if STR was higher than 70%.

Sperm morphology was evaluated in two replicates (100 sperm cells per replicate) using the Sperm Class Analyzer (SCA) software (Microptic; Spain) and a phase-contrast microscope (Olympus BX41; Japan) at 200× magnification. Each spermatozoon was classified as normal or abnormal. Abnormal sperm cells were subsequently classified as with tail abnormalities (including folded and coiled tails), isolated heads, or cytoplasmic droplets (including proximal and distal droplets).

### Oxygen consumption

Oxygen consumption in sperm was assessed using a SensorDish Reader system (PreSens Gmbh; Regensburg, Germany). Samples were diluted by mixing 150 μL of semen sample with 850 μL of PBS, and then transferred onto Oxodish OD24 plates before sealing them with Parafilm. Plates were incubated in the reader system at 38 °C, and oxygen consumption was measured every 30 s for 4 h. Oxygen consumption rate was normalized against the total number of sperm per sample and evaluated in two technical replicates.

### Flow cytometry analysis

Several sperm functionality parameters were measured using a CytoFLEX flow cytometer (Beckman Coulter; CA, USA), equipped with 637, 488 and 405 nm lasers. These parameters included viability, acrosome membrane integrity, levels of chromatin (de)condensation, membrane lipid disorder, intracellular calcium levels, total reactive oxygen species (ROS) and intracellular superoxide radicals. The evaluation of all these sperm function parameters has been described in detail elsewhere [19–23]. Briefly, sperm viability was assessed by staining samples with SYBR-14 (32 nmol/L, excited by 488-nm laser and detected through the FITC channel: 525/40 nm) and propidium iodide (PI) (7.5 µmol/L, excited by 488-nm laser and detected through the PC5.5 channel: 690/50 nm) at 38 ºC for 15 min in the dark. The percentage of viable (PI^−^) and green-stained sperm (SYBR-14^+^) was used to calculate sperm viability. Acrosome integrity was evaluated by staining samples with Peanut agglutinin (PNA) (1.17 µmol/L, excited by 488-nm laser and detected through the FITC channel: 525/40 nm) and PI (5.6 µmol/L) at 38 ºC for 10 min. Chromatin (de)condensation was evaluated after diluting sperm samples 1:1 (v:v) in 2× McIlvine solution to a final concentration of 20 × 10^6^ sperm/mL, and staining with Chromomycin A3 (CMA3) (12.5 µg/mL, excited by 405-nm laser and detected through the Violet610 channel: 610/20 nm) and Yo-Pro-1 (0.2 µmol/L, excited by 488-nm laser and detected through the FITC channel: 525/40 nm) at room temperature for 20 min in the dark. Membrane lipid disorder was assessed through staining with Merocyanine 540 (M540) (10 nmol/L, excited by 488-nm laser and detected through the ECD channel: 610/20 nm) and Yo-Pro-1 (31.25 nmol/L) at 38 ºC for 15 min in the dark. Intracellular calcium levels were evaluated by staining sperm samples with Fluo-3-AM (1.2 µmol/L, excited by 488-nm laser and detected through the FITC channel: 525/40 nm) and PI (5.6 µmol/L) for 10 min at 38 ºC. Total reactive oxygen species were determined by staining with 2',7'-dichlorodihydrofluorescein diacetate (H_2_DCFDA) (100 µmol/L, excited by 488-nm laser and detected through the FITC channel: 525/40 nm) and PI (5.6 µmol/L) for 20 min at 38 ºC. Finally, intracellular superoxide radicals were measured by incubating samples with Hydroethidine (HE) (5 µmol/L, excited by 488-nm laser and detected through the PE channel: 585/42 nm) and Yo-Pro-1 (31.25 nmol/L) for 20 min at 38 ºC. CytExpert v.2.4 software was used to perform data analysis, following the recommendations of the International Society for Advancement of Cytometry (ISAC) [24].

### Evaluation of miRNA expression

Candidate miRNAs were selected based on their association with major genes known to be expressed in sperm cells, epididymis and/or testis [7, 25–27]. A decision was ultimately made to include ten miRNAs for quantification: bta-miR-10a, bta-miR-10b, bta-miR-138, bta-miR-146b, bta-miR-19b, bta-miR-26a, bta-miR-34a, bta-miR-449a, bta-miR-495 and bta-miR-7. Additional file 1 describes the main characteristics of the selected miRNAs candidates.

#### RNA isolation and quantification

Prior to RNA isolation, semen samples were processed through somatic cell lysis (SCL) according to the method reported by Goodrich et al*.* [28], with the aim to remove any somatic cell contaminant. In brief, sperm samples were washed with 1 mL of PBS at 1,000 × *g* for 10 min and pellets were resuspended in 1 mL of SCL buffer (0.1% sodium dodecyl sulfate and 0.5% Triton X-100 in milliQ water) and maintained on ice for 30 min. After 20 min, an examination under a phase-contrast microscope was performed to confirm the absence of somatic cells. Samples were finally centrifuged at 1,000 × *g* for 10 min and immediately subject to RNA isolation.

Total RNA isolation was performed following the RNeasy Mini Kit (QIAGEN; CA, USA) isolation protocol with few modifications. Briefly, samples were washed in PBS and centrifuged twice at 1,000 × *g* for 10 min. Thereafter, 600 μL of the RNeasy lysis buffer (RLT) and 10 μL of dithiothreitol (DTT) were added to each sample; the homogenate was incubated at 56 ºC for 15 min. Ethanol 70% was then added to lysates, and samples were transferred onto a RNeasy Mini spin column. Afterwards, lysates were treated with DNase I (Thermo Fisher Scientific; MA, USA) and subsequently washed three times to remove DNase and purify the isolated RNA. The resulting RNA samples were eluted with 15 µL of RNase free water (Thermo Fisher Scientific; MA, USA) and maintained at −80 ºC until miRNA analysis.

The quantity and quality of RNA, measured as RNA concentration and purity (260/280 nm ratio), were determined with an Agilent Epoch spectrophotometer (Mettler Toledo; Giessen, Germany). Another quality control analysis verified the lack of ribosomal RNA (rRNA) in RNA-isolated samples using an Agilent RNA 6000-Nano chip and the Agilent 2100 Bioanalyzer (Agilent Technologies; CA, USA), according to manufacturer’s instructions. A positive control (RNA isolated from Jurkatt cells using the same protocol) was included to verify that the method conducted for RNA isolation preserved nucleic acid integrity, using the RNA integrity number (RIN) as an indicator.

#### RT-qPCR and data analysis

The expression of the ten miRNA candidates was evaluated using the TaqMan Advanced miRNA Assays kit (Thermo Fisher Scientific; MA, USA) and a Reverse Transcription quantitative PCR (RT-qPCR) thermal cycler 7900 (Life Technologies; MA, USA). Briefly, 10 ng of total RNA was used to perform the poly(A) tailing reaction (45 min at 37 ºC, 10 min at 65 ºC and holding at 4 ºC). Immediately, samples were submitted to the adaptor ligation reaction (60 min at 16 ºC and holding at 4 ºC). The TaqMan Advanced miRNA cDNA Synthesis Kit (Thermo Fisher Scientific; MA, USA) was used for preparing cDNA templates using the following thermal cycling profile (42 ºC for 15 min and 85 ºC for 5 min), and a miR-Amp reaction protocol was subsequently employed to amplify the amount of cDNA (conditions: 95 ºC for 5 min, 14 cycles of 95 ºC for 3 s and 60 ºC for 30 s, 10 min at 99 ºC, and a final holding step at 4 ºC). RT-qPCR analyses were performed in duplicates using 5 µL of the pre-amplified product (conditions: 95 ºC for 20 s, and 40 cycles of 95 ºC for 3 s and 60 ºC for 30 s, and a final holding step at 4 ºC).

The *C. elegans* cel-miR-39 (Thermo Fisher Scientific; MA, USA) was used as a spike-in control. This miRNA is a sequence typically used for the analysis of samples with low RNA quantity (such as non-coding RNAs), and extensively employed for the normalization of miRNA expression [29]. The spike-in represents a control with a known concentration/copy level that allows the generation of a standard curve and normalization. The expression level of each miRNA was measured with the cycle threshold (Ct), defined as the number of cycles required for the fluorescent signal to cross the threshold (i.e., exceeding the background level). The arithmetic mean of the Ct duplicates was used and normalized Cts (or ΔCt) were then calculated with the following formula: Ct miRNA value − Ct spike-in value. The 2^–∆∆Ct^ method (Livak-Schmittgen method [30]) was applied to explore the differential expression (DE) of miRNAs between the two groups of bulls (high fertility vs. subfertility).

#### Predicted targets and Gene Ontology analysis

The potential targets of miRNAs that are associated with sperm functionality or fertility potential were predicted using the TargetScan v7.1 software (https://www.targetscan.org/cgi-bin/targetscan/vert_71) [31]. Only transcripts with conserved sites were included in the analysis. Moreover, the enrichment of biological processes in which these genes could be involved was evaluated through PANTHER v17.0 (http://pantherdb.org/) using the Fisher’s exact test [32]. The total bovine genome was used as a background and only those biological processes with *P*-values < 0.05 were considered to be significantly enriched after applying a False Discovery Rate (FDR) correction. To summarize and visualize the significant GO terms the corresponding processes were submitted to a REVIGO analysis (http://revigo.irb.hr/) [33].

### Statistical analysis

Data were analyzed using IBM SPSS Statistics 28.0 (IBM; NY, USA) and plotted with GraphPad Prism v8 (GraphPad; MA, USA). Descriptive data are presented as mean ± standard deviation (SD). All variables subject to statistical inference were first checked for normal distribution (Shapiro–Wilk test) and homogeneity of variances (Levene test). T-tests or U-Mann Whitney tests were run to compare the two fertility groups (independent variable) depending on the fulfilment of normality and homoscedasticity assumptions in each sperm quality/functionality variable (dependent variables: total and progressive motility, VCL, VSL, VAP, LIN, STR, WOB, ALH, BCF, normal morphology, cytoplasmic droplets, tail abnormalities, and isolated heads). Nonparametric Mann–Whitney tests were used to determine statistical differences in miRNA expression (dependent variable) between fertility groups (independent variable). Additionally, the Spearman's rank coefficient test was used to evaluate correlations between sperm motility/kinematics, morphology, flow cytometry parameters and the level of expression of miRNAs. Only rho coefficients > 0.45 and with a significance level ≤ 0.01 are referred in the results section, as only these ones were considered to have a plausible biological role. The level of significance was set at *P* < 0.05 (two-tailed).

## Results

A total of 29 sperm samples from 29 individual bulls (SF [*n* = 15] and HF [*n* = 14]) were used in the analysis. Bulls were classified based on their NRR (median, interquartile range, IQR) into two groups: high fertility (HF: 40.5, 40.2–42.0) and subfertility (SF: 37.7, 36.5–38.9). The percentage of progressively motile sperm was significantly higher in the HF (31.75% ± 8.41%) than in the SF group (22.93% ± 8.90%) (*P* = 0.011). No other significant differences were found for any of the morphology, motility, and kinematics parameters (Table 1).

**Table 1:** Descriptive data of the population studied: Motility, kinematic parameters and morphology parameters for each fertility group; subfertility group (SF) and high fertility group (HF)

		**Motility and kinematics parameters**										**Morphology parameters**			
**Animal code**	**NRR**	**Total motility, %**	**Progressive motility, %**	**VCL, µm/s**	**VSL, µm/s**	**VAP, µm/s**	**LIN, %**	**STR, %**	**WOB, %**	**ALH, µm**	**BCF, Hz**	**Normal morphology, %**	**Cytoplasmic droplets, %**	**Tail abnormalities, %**	**Isolated head, %**
Subfertility (SF) group															
S13	33.33	41.79	11.30	39.19	17.01	21.27	43.41	79.98	54.27	2.38	9.75	93.60	0.45	3.66	2.32
S25	34.58	33.62	19.59	68.26	35.05	42.02	51.07	83.61	61.13	2.53	12.72	86.40	0.00	11.17	2.43
S11	36.25	44.65	18.10	62.77	21.84	29.91	34.77	73.00	47.63	2.97	9.11	88.85	0.00	3.89	5.32
S16	36.40	26.39	14.67	62.14	30.88	37.01	49.66	83.10	59.53	2.18	11.49	92.45	0.00	7.59	0.00
S26	36.52	39.21	19.70	72.70	28.15	37.26	38.71	75.53	51.25	3.17	9.21	90.10	0.00	6.94	1.99
S9	36.86	52.02	25.65	75.20	40.76	47.82	54.22	85.22	63.63	2.89	12.49	85.55	0.50	7.98	3.99
S17	37.60	42.54	21.79	59.51	30.01	34.62	49.98	86.24	57.91	2.76	12.64	84.80	0.00	11.86	3.33
S14	37.65	62.18	31.87	81.61	48.92	55.08	59.94	88.83	67.48	3.25	12.51	91.35	0.00	6.22	1.47
S21	38.25	25.33	16.33	89.56	46.57	52.18	51.90	89.13	58.22	3.64	12.37	94.00	0.00	5.00	1.00
S29	38.43	20.62	14.11	85.78	45.93	50.58	53.52	90.84	58.93	3.36	13.69	84.15	0.91	10.78	3.36
S2	38.84	55.12	21.78	71.05	37.88	43.61	53.22	86.69	61.32	3.29	11.71	88.99	0.96	5.77	2.36
S19	38.98	69.68	43.73	96.46	52.73	58.57	54.77	90.13	60.77	3.65	12.95	88.70	1.00	6.50	1.95
S5	39.11	26.06	20.06	79.64	46.61	51.58	58.52	90.35	64.77	2.29	13.43	94.30	0.50	1.83	3.38
S22	39.34	48.33	29.38	91.40	45.21	52.83	49.46	85.59	57.79	3.59	11.19	88.95	0.48	5.46	2.83
S4	39.34	48.13	35.89	108.85	73.67	77.85	67.56	94.58	71.42	3.29	14.21	89.85	0.36	6.17	3.63
Mean (SD)	37.43 (1.79)	42.38 (14.24)	22.93 (8.90)	76.27 (17.21)	40.08 (13.98)	46.15 (13.56)	51.38 (8.12)	85.52 (5.84)	59.74 (5.98)	3.01 (0.50)	11.96 (1.57)	89.47 (3.28)	0.34 (0.38)	6.72 (2.83)	2.62 (1.30)
High fertility (HF) group															
S23	39.51	60.45	32.88	79.56	40.36	46.65	50.69	86.46	58.61	3.33	11.50	86.90	0.00	10.86	2.27
S3	40.03	28.60	17.44	94.55	47.96	54.77	50.44	87.21	57.80	3.71	12.09	92.00	0.00	6.00	0.50
S24	40.13	58.31	37.34	86.15	46.85	54.21	54.23	86.40	62.76	2.92	12.65	88.95	1.43	7.13	1.96
S10	40.13	61.46	41.24	91.84	49.21	55.09	53.49	89.26	59.92	3.41	13.38	89.15	2.95	3.94	3.46
S1	40.29	30.91	20.38	95.74	50.28	55.99	52.40	89.69	58.41	3.92	12.16	94.00	0.00	4.50	1.00
S28	40.33	41.24	24.47	67.02	31.40	37.89	46.26	82.01	56.10	2.34	11.86	84.35	0.00	11.40	2.84
S15	40.37	60.48	35.22	86.43	48.19	53.97	55.72	89.28	62.41	3.27	13.72	85.45	0.00	11.63	2.44
S18	40.60	74.47	48.61	104.17	62.27	73.69	59.75	84.48	70.72	3.21	11.77	91.70	1.47	4.83	1.50
S7	41.06	43.40	29.65	108.27	61.15	67.77	56.48	90.24	62.59	3.67	13.33	91.85	0.47	3.55	2.05
S27	41.34	51.41	31.45	75.89	41.28	47.30	54.50	87.28	62.42	2.69	13.25	90.15	0.99	3.94	4.43
S12	42.28	56.44	40.44	108.14	65.11	71.72	60.14	90.62	66.37	3.34	14.54	81.45	1.92	9.01	6.68
S20	42.34	56.89	27.65	73.56	35.14	41.91	47.84	83.88	57.03	3.36	10.52	89.35	0.48	3.40	5.32
S8	42.94	51.73	28.83	54.89	26.55	31.69	48.42	83.79	57.78	2.25	11.64	86.10	1.49	10.45	1.98
S6	43.48	52.39	28.91	60.90	26.87	33.25	43.98	80.64	54.52	2.42	11.70	91.75	1.45	6.36	0.45
Mean (SD)	41.06 (1.23)	52.01 (12.41)	31.75 (8.41)	84.79 (16.99)	45.19 (12.46	51.85 (13.20)	52.45 (4.82)	86.52 (3.17)	60.53 (4.35)	3.13 (0.53)	12.44 (1.08)	88.80 (3.53)	0.90 (0.92)	6.93 (3.13)	2.63 (1.80)
*P*-value	** < 0.001**^**b**^	0.063^**a**^	**0.011**^**a**^	0.191^**a**^	0.310^**a**^	0.262^**a**^	0.672^**a**^	0.747^**b**^	0.687^**a**^	0.548^**a**^	0.357^**a**^	0.599^**a**^	0.134^**b**^	0.853^**a**^	0.986^**a**^

Data are presented as mean (standard deviation; SD). Data were checked for normal distribution (Shapiro–Wilk test) and homogeneity of variances (Levene test) prior to statistical analysis. Independent T-tests ^a^ or U-Mann Whitney tests ^b^ were conducted to determine statistical differences between SF and HF groups with a *P*-value < 0.05 depending on the normality and homoscedasticity assumptions. Significant values are bolded

*Abbreviations*: *ALH* Mean amplitude of the lateral oscillatory movement of the sperm head around the mean trajectory, *BCF* Number of sperm head lateral oscillatory movements around the mean trajectory per unit of time, *HF* High fertility group, *LIN* Linearity coefficient (LIN = VSL/VCL × 100), *NRR* Non-return rates, *RIN* RNA integrity number, *rRNA* Ribosomal RNA, *S* Svedberg sedimentation coefficient, *SD* Standard deviation, *SF* Subfertility group, *STR* Straightness coefficient (STR = VSL/VAP × 100), *VAP* Mean sperm trajectory per second, *VCL* Instantaneous sequential progression along the trajectory, *VSL* Straight sperm trajectory per second, *WOB* Wobble coefficient (WOB = VAP/VCL × 100)

RNA concentration and purity (260/280 nm ratio) (mean ± SD) of the 29 samples were 99.3 ± 84.6 ng/µL and 1.97 ± 0.72, respectively (Additional file 2). No significant differences in RNA concentration or purity were observed between SF and HF groups. Moreover, bioanalyzer results showed no ribosomal RNA in isolated samples, confirming the lack of RNA from somatic cells (Additional file 3).

**Fig. 1:**
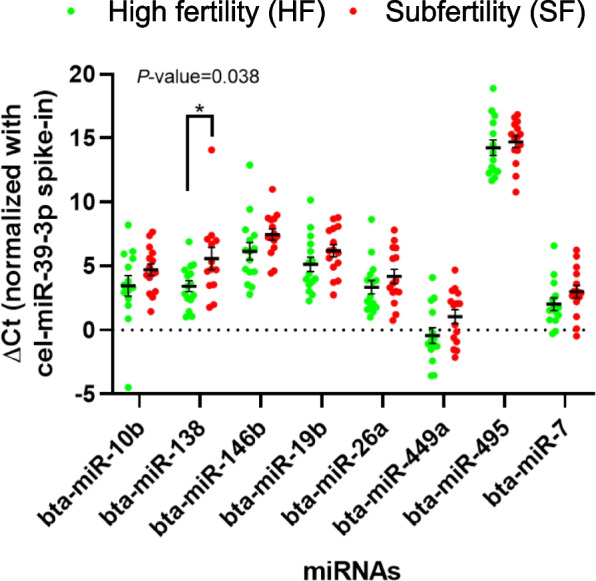
Mean ± SEM of normalized Ct values (ΔCt) of the miRNAs present in sperm from bulls of high fertility (HF) and subfertility (SF). 2^–∆∆Ct^ method and nonparametric Mann-Whiney test were used to determine statistical differences between groups

In terms of miRNA presence or absence, eight out of ten miRNAs (bta-miR-10b, bta-miR-138, bta-miR-146b, bta-miR-19b, bta-miR-26a, bta-miR-449a, bta-miR-495, and bta-miR-7) were consistently detected in bovine sperm. In contrast, neither bta-miR-10a nor bta-miR-34a were found. Remarkably, one of the miRNAs (bta-miR-138) was downregulated (*P* = 0.038) in the sperm of the SF group compared to the HF group (ΔCt HF = 22.76 ± 1.45 vs. ΔCt SF = 24.40 ± 3.87) (Fig. 1).

**Fig. 2:**
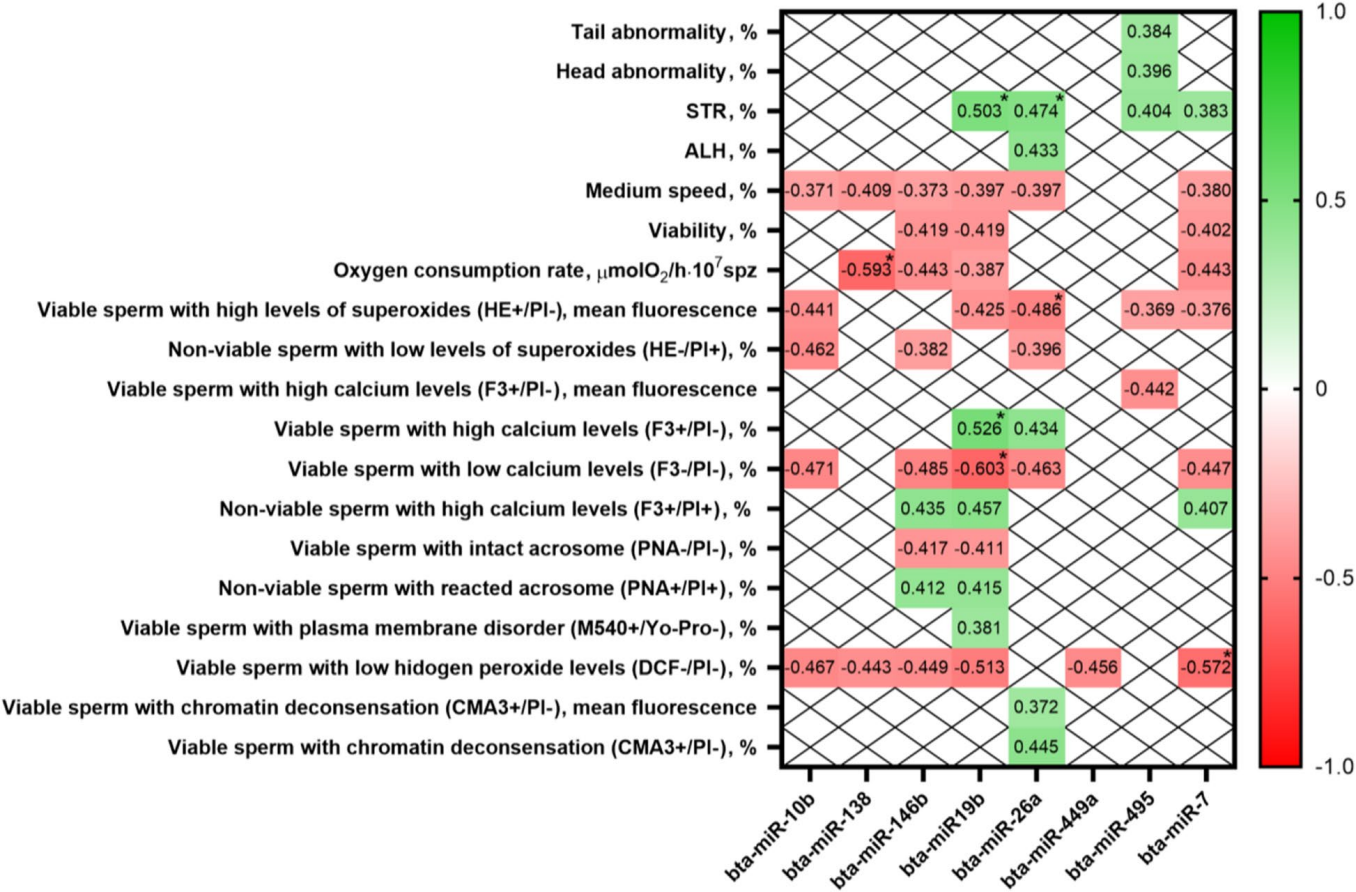
Heat map of statistically significant Spearman correlation coefficients (rho values) between detected miRNAs and sperm functionality parameters. All correlations displayed here were significant (*P* ≤ 0.05). Correlation coefficients containing an asterisk (*) were significant at *P* ≤ 0.01 (2-tailed). Mean fluorescence represents the mean of the fluorescence intensity of all measurements. Abbreviations: M540, Merocyanine 540; ALH, amplitude of lateral head displacement; CMA3, chromomycin A3; DCF, 2,7-dichlorofluorescein; F3, Fluo-3-pentaacetoxymethyl ester; HE, hydroethidine; PI, propidium iodide; PNA, lectin peanut agglutinin; STR, straightness of trajectory

Further analyses examined the potential correlations between miRNA expression in sperm and sperm quality and functionality outcomes. All correlation coefficients found to be statistically significant (*P* < 0.05) are shown in Fig. 2. As aforementioned, only rho correlation coefficients > 0.45 and with a significance level ≤ 0.01 were considered to be relevant. In brief and in addition to being associated with fertility potential measured by NRR, the relative content of bta-miR-138 was found to be negatively correlated with sperm oxygen consumption (rho = −0.593, *P* < 0.01). The bta-miR-19b expression was positively correlated with the percentages of straightness (a kinematic parameter) and of viable sperm with high calcium levels (rho = 0.503, *P* < 0.01; and 0.526, *P* < 0.01, respectively), and negatively correlated with the percentage of viable sperm with low calcium levels (rho = −0.603, *P* < 0.01). Moreover, the expression of bta-miR-26a was seen to be positively correlated with the percentage of straightness (rho = 0.474, *P* < 0.01), and negatively with the proportion of viable sperm with high levels of superoxides (rho = −0.486, *P* < 0.01). Finally, the relative expression of bta-miR-7 was found to be negatively correlated with the percentage of viable sperm with low ROS levels (rho = −0.572, *P* < 0.01).

## Discussion

### Principal findings

The present research confirmed the presence of eight out of the ten miRNA candidates in bovine sperm and provided evidence about the role of bta-miR-138 in fertilization and post-fertilization events, as the expression of this miRNA was upregulated in sperm giving rise to higher fertility rates. The study, which used a standardized and optimized methodology to isolate and quantify miRNA from bovine sperm, also identified three other miRNAs (bta-miR-19b, bta-miR-26a and bta-miR-7) whose expression was positively or negatively correlated with sperm functionality parameters.

### Results in context

Accumulating evidence supports the relevance of non-coding RNA species for the regulation of cellular phenotypes/characteristics and reproductive processes in both humans and farm animals [7, 34]. Given the relative simplicity of determining the presence of specific miRNA species in biological samples (e.g., blood, semen, tissues, etc.), there is a rising interest in exploring if this class of non-coding RNAs is related to fertilization-related outcomes, including sperm quality, fertilization success and embryo survival [12, 13, 35].

The current study shows, for the first time, a consistent positive relationship between the expression of bta-miR-138 in bovine sperm and fertilizing ability. These results in cattle are in line with those of Salas-Huetos et al. [12, 13], who showed in two recent systematic reviews consistent associations between infertility and the expression of some miRNAs in reproductive cells/tissues.

Other authors also described that the expression of miR-138 and of other members of the miR-34-5p/449-5p family significantly differs between in-vivo and in-vitro produced bovine embryos [35], indicating that these miRNAs are a key component of embryo implantation, pregnancy and early embryogenesis. Notwithstanding the present study supports the use of bta-miR-138 as a potential biomarker of sperm fertility, several questions remain unanswered at present and future analyses are therefore recommended. This work also found that the expression of the aforementioned miRNA was significantly and negatively correlated with oxygen consumption, which is known to be associated with sperm metabolism [36], suggesting that the less oxygen consumption the less metabolic activity in bovine sperm. In fact, the basal metabolic activity of sperm appears to influence their function, even beyond fertilization [37]. It is possible, therefore, that a lower metabolic rate indicates that sperm do not hyperactivate prematurely, which would be associated with a greater fertilizing ability [38]. Media surrounding mammalian sperm must have a specific composition of ions and metabolites in order for cells to undergo capacitation, trigger the acrosome reaction and fertilize the oocyte, among other physiological processes. The different substrate and oxygen concentrations that sperm cells encounter in their journey along the male and female tracts towards the oocyte influence metabolic pathways, mainly oxidative phosphorylation (Oxphos) and glycolysis [39]. While Oxphos and glycolysis seem to be balanced in cattle [40], any insight into the relationship of sperm miRNAs with sperm metabolism and fertility deserves attention. In this study, although the levels of bta-miR-7 were not found to be associated with fertility potential, they were observed to be negatively correlated with low hydrogen peroxide levels, which could be linked to sperm metabolic pathways [41]. Further research should thus address if variations in the relative levels of sperm miRNAs are related to changes in metabolism, which could eventually have an impact on fertility. Any further effort should, nevertheless, be able to identify the confounding factors that may affect the relationships described.

While other miRNAs whose presence was analyzed in bovine sperm were not found to be associated with their fertilizing ability, they were observed to be significantly correlated with different functional sperm characteristics, which could ultimately have the potential to impact fertility. Interestingly, the expression trend of the analyzed miRNAs was the same in each sperm variable. For example, when STR was evaluated, all correlations with the expression of miRNAs were positive (bta-miR-19b, and -26a). These findings reinforce the notion that the miRNAs present in sperm may have a specific role, and that sperm-borne miRNAs are not only remnants of spermatogenesis [42]. On the other hand, very recently, Donnellan et al*.* sought to describe the content of both mRNAs and miRNAs in bovine sperm, and found that two of the miRNAs identified in the present study (bta-miR-449a and bta-miR-7) were among the ones most highly expressed [43]. Even though the aforementioned study only reported that these two miRNAs are highly expressed in this type of cells, the present work also supports that not only are these miRNAs consistently present in bovine sperm, but they could also be somehow related to sperm functionality as in the case of miR-7 with regard to low hydrogen peroxide levels.

### Strengths and limitations

The most significant strength of this research is that, to the best of the authors’ knowledge, it describes, for the first time in cattle and through a standardized and validated miRNA isolation and quantification methodology, that bta-miR-138 may be linked to bull fertility. In addition, this study indicates that the expression of this miRNA is related to some functional sperm parameters (e.g., DNA condensation, acrosome development) and post-spermatogenesis events (e.g., sperm capacitation, acrosome reaction), and supports that it could be used a fertility biomarker.

Whilst the conducted analysis provides valuable information about the presence of particular miRNAs and their relative associations with specific sperm characteristics and fertility outcomes, there are several limitations and uncertainties that should be taken into consideration when interpreting the results. First of all, this study is largely descriptive and functional validation experiments using microinjection of bta-miR-138 antisense sequence miRNAs into in vitro fertilized oocytes are warranted to confirm the results. Indeed, bovine sperm may contain as many as 13,000 transcripts, and miRNAs (about 1,000 in bovine) can be associated anywhere from several hundred to over a thousand of mRNA targets [44]. This allows for a high number of assumptions about their possible associations, which may also be defined by relative miRNA and mRNA abundance, both during spermatogenesis as well as post-fertilization. Second, the high fertility potential of the animals involved made it difficult to establish groups of fertile and infertile animals; instead, the two groups, which consistently showed differences in their motility and NRR, were actually of subfertility vs. good fertility. This could have masked the relevance of the other miRNAs examined, but should not affect the collected evidence about the role of bta-miR-138. Future studies should also confirm these results by evaluating how miRNAs levels vary when groups established on the basis of sire conceptions rates are compared. Third, despite the fact that this study found consistent relationships between miRNAs expression and sperm functionality parameters, frozen-thawed sperm samples were not of optimal quality. The use of fresh sperm could give rise to different associations beyond those reported herein. Lastly, due to the relatively small sample size, some of the findings should be interpreted with caution until validated/reconfirmed in further studies, including the fact that one of the most abundant sperm miRNAs (bta-miR-34a) was not detected in 23 out of 29 of the samples. This definitely deserves special attention in other similar studies. The current study, in short, advises replicating the research using animals with very low fertility rates; yet, the authors are aware of the difficulties of recruitment.

## Conclusions

In conclusion, this report indicates the presence of eight miRNAs in bovine sperm (bta-miR-10b, bta-miR-138, bta-miR-146b, bta-miR-19b, bta-miR-26a, bta-miR-449a, bta-miR-495, and bta-miR-7) and reveals that, in cattle, bta-miR-138 is linked to bull fertility. Functional validation studies are, however, required to confirm these results. Apart from paving the way to study through which specific mechanism this miRNA plays such a role, this research supports its use as a fertility biomarker. The fact that three other miRNAs (bta-miR-19b, bta-miR-26a and bta-miR-7) were found to be correlated to sperm functional variables suggests that their relative expression could reflect what occurs during spermatogenesis and spermiogenesis, and would also warrant further research in a larger number of samples.

### Supplementary Information

**Additional file 1.** Characteristics of miRNA candidates in bovine. ^a^Information extracted from TargetScan website (last accession 13/04/2023): https://www.targetscan.org/vert_71/. ^b^Information extracted from: http://genome-euro.ucsc.edu/, assembly Apr. 2018 (ARS-UCD1.2/bosTau9).

**Additional file 2.** RNA quantity and quality data for each fertility group, determined through Epoch spectrophotometer and Bioanalyzer. Data are presented as mean (standard deviation; SD). Data were checked for normal distribution and homogeneity of variances prior to statistical analysis. U-Mann Whitney test was run to compare SF and HF groups with a *P*-value < 0.05. Abbreviations: HF, high fertility group; RIN, RNA integrity number; rRNA, ribosomal RNA; S, svedberg sedimentation coefficient; SD, standard deviation; SF, subfertility group.

**Additional file 3.** Output of Bioanalyzer 2100 analysis. **A** Representative electropherogram of RNA isolated from Jurkatt cells and used as a positive control. **B** Representative electropherogram of RNA isolated form bovine sperm cells. Abbreviations: FU, fluorescence units; nt, nucleotides; RIN, RNA integrity number; rRNA, ribosomal RNA; S, svedberg sedimentation coefficient.

**Additional file 4.** Target transcripts of the miRNAs candidates associated to fertility potential (bta-miR-138) or sperm function (bta-miR-7, bta-miR-19b, and bta-miR-26a). Information was extracted from TargetScan website (last accession 13/04/2023): https://www.targetscan.org/vert_71/.

**Additional file 5.** Gene Ontology (GO) analysis of the miRNAs-targets for bta-miR-138, bta-miR-7, bta-miR-19b and bta-miR-26a. Analyses were conducted with PANTHER 17.0 (http://pantherdb.org/) using Fisher’s exact test and False Discovery Rate (FDR) correction.

**Additional file 6.** REVIGO clustering of Gene Ontology analysis of the predicted target genes for bta-miR-138. The axes in the plot have no intrinsic meaning. REVIGO uses Multidimensional Scalingto reduce the dimensionality of a matrix of the GO terms pairwise semantic similarities.

## Data Availability

The datasets used and/or analyzed during the current study are available from the corresponding author on reasonable request.

## Abbreviations

ΔCt: Normalized Cts
ALH: Lateral head displacement
BCF: Frequency of head displacement
CMA3: Chromomycin A3
DCF: 2,7-Dichlorofluorescein
DE: Differential expression
DTT: Dithiothreitol
F3: Fluo-3-pentaacetoxymethyl ester
FU: Fluorescence units
H_2_DCFDA: 2',7'-Dichlorodihydrofluorescein diacetate
HE: Hydroethidine
HF: High fertility
ISAC: International Society for Advancement of Cytometry
LIN: Linearity
M540: Merocyanine 540
miRNA: MicroRNA
NRR: Non-return rates
Nt: Nucleotides
Oxphos: Oxidative phosphorylation
PI: Propidium iodide
PNA: Peanut agglutinin
RIN: RNA integrity number
RLT: RNeasy lysis buffer
ROS: Reactive oxygen species
rRNA: Ribosomal RNA
RT-qPCR: Reverse transcription quantitative PCR
S: Svedberg sedimentation coefficient
SCA: Sperm class analyzer
SCL: Somatic cell lysis
SD: Standard deviation
SF: Subfertility
STR: Straightness
TFR: Total fertility range
VAP: Average path velocity
VCL: Curvilinear velocity
VSL: Straight-line velocity
WOB: Oscillation
