# Supplementary material for: Expression of miR-138 in cryopreserved bovine sperm is related to their fertility potential
Source: J Anim Sci Biotechnol. 2023 Sep 20;14:129. doi: 10.1186/s40104-023-00909-1 (PMC10510164; doi:10.1186/s40104-023-00909-1)
Supplement: Supplementary file 1 — Additional file 1. Characteristics of miRNA candidates in bovine. aInformation extracted from TargetScan website (last accession 13/04/2023): https://www.targetscan.org/vert_71/. bInformation extracted from: http://genome-euro.ucsc.edu/, assembly Apr. 2018 (ARS-UCD1.2/bosTau9). [file 40104_2023_909_MOESM1_ESM.docx]

**Additional File 1.**

| **bta-miRNA** | **Localization and sequence** | **Number of potential target transcripts with conserved sites ^a^** | **Main genes near the region ^b^** | **Bull reproductive tissue where the miRNA has been previously described and main reference** |
| --- | --- | --- | --- | --- |
| miR-7 | chr21: 20048987-20049084  UGGAAGACUAGUGAUUUUGUUGUU | 532 | LOC508153 | Epididymis [25] |
| miR-10a | chr19: 38713901-38714009  UACCCUGUAGAUCCGAAUUUGUG | 299 | TBX21/ LNPK | Epididymis [25] |
| miR-10b | chr2: 20876466-20876564  UACCCUGUAGAACCGAAUUUGUG | 299 |  | Epididymis [25] |
| miR-19b | chr12: 66421815-66421901  UGUGCAAAUCCAUGCAAAACUGA | 1,234 | GPC5 | Epididymis [25] |
| miR-26a | chr22: 11513974-11514063  UUCAAGUAAUCCAGGAUAGGCU | 945 | DLEC1 | Epididymis [25] |
| miR-34a | chr16: 45359550-45359656  UGGCAGUGUCUUAGCUGGUUGU | 668 | ERRFI1 | Epididymis [25] |
| miR-138 | chr22: 14939860-14939956  AGCUGGUGUUGUGAAUCAGGCCG | 659 | ACKR2/CPNE2/ HERPUD1 | Testis [27] |
|  | chr18: 25049218-25049300  AGCUGGUGUUGUGAAUCAGGCCG |  |  |  |
| miR-146b | chr26: 23050499-23050604  UGAGAACUGAAUUCCAUAGGCUGU | 248 | CUEDC2 | Testis [27] |
| miR-449a | chr20: 24047150-24047237  UGGCAGUGUAUUGUUAGCUGGU | 668 | GZMA/ GZMK | Testis and epididymis [25,27] |
| miR-495 | chr21: 67851608-67851688  AAACAAACAUGGUGCACUUCUU | 821 | TNFAIP2 | Testis [27] |
