# Supplementary material for: Expression of miR-138 in cryopreserved bovine sperm is related to their fertility potential
Source: J Anim Sci Biotechnol. 2023 Sep 20;14:129. doi: 10.1186/s40104-023-00909-1 (PMC10510164; doi:10.1186/s40104-023-00909-1)
Supplement: Supplementary file 2 — Additional file 2. RNA quantity and quality data for each fertility group, determined through Epoch spectrophotometer and Bioanalyzer. Data are presented as mean (standard deviation; SD). Data were checked for normal distribution and homogeneity of variances prior to statistical analysis. U-Mann Whitney test was run to compare SF and HF groups with a P-value < 0.05. Abbreviations: HF, high fertility group; RIN, RNA integrity number; rRNA, ribosomal RNA; S, svedberg sedimentation coefficient; SD, standard deviation; SF, subfertility group. [file 40104_2023_909_MOESM2_ESM.docx]

**Additional File 2.**

|  | **Epoch spectrophotometer** | | **Bioanalyzer** | | |
| --- | --- | --- | --- | --- | --- |
| **Animal code** | **Concentration (ng/µL)** | **Purity (260/280 nm ratio)** | **Concentration (ng/µL)** | **RIN (28S and 18S rRNA integrity)** | |
| **Subfertility (SF) group** | | | | |  |
| S13 | 95.8 | 1.88 | 30.0 | 1.00 | |
| S25 | 241.8 | 1.91 | 145.0 | 2.20 | |
| S11 | 13.6 | 1.76 | 13.0 | 1.00 | |
| S16 | 362.8 | 1.62 | 89.0 | 2.20 | |
| S26 | 38.0 | 2.01 | 48.0 | 1.10 | |
| S9 | 101.2 | 1.91 | 96.0 | 1.90 | |
| S17 | 72.7 | 1.92 | 23.0 | 1.10 | |
| S14 | 11.8 | 1.54 | 1.0 | NA | |
| S21 | 135.0 | 1.92 | 129.0 | 2.40 | |
| S29 | 140.7 | 1.92 | 91.0 | 1.50 | |
| S2 | 11.5 | 2.07 | 11.0 | 1.00 | |
| S19 | 70.1 | 1.94 | 42.0 | 1.80 | |
| S5 | 185.7 | 1.89 | 175.0 | 2.40 | |
| S22 | 3.9 | 5.53 | 223.0 | 2.30 | |
| S4 | 4.5 | 2.10 | 3.0 | NA | |
| **SF; mean (SD)** | 99.27 (102.23) | 2.18 (0.28) | 85.77 (18.53) | 1.68 (0.16) | |
| **High fertility (HF) group** | | | | |  |
| S23 | 128.7 | 1.94 | 94.0 | 1.70 | |
| S3 | 87.8 | 1.89 | 60.0 | 2.70 | |
| S24 | 198.7 | 1.91 | 72.0 | 2.10 | |
| S10 | 75.8 | 1.73 | 39.0 | 1.90 | |
| S1 | 35.1 | 1.84 | 44.0 | 1.20 | |
| S28 | 102.2 | 1.69 | 46.0 | 2.50 | |
| S15 | 3.1 | 0.82 | 6.0 | NA | |
| S18 | 40.7 | 1.87 | 8.0 | NA | |
| S7 | 80.7 | 1.89 | 28.0 | 1.00 | |
| S27 | 143.6 | 1.92 | 98.0 | 1.70 | |
| S12 | 58.0 | 1.89 | 34.0 | 1.00 | |
| S20 | 111.0 | 1.91 | 45.0 | 2.20 | |
| S8 | 244.8 | 1.94 | 146.0 | 2.30 | |
| S6 | 81.1 | 1.86 | 35.0 | 1.10 | |
| **HF; mean (SD)** | 99.38 (64.39) | 1.87 (0.02) | 61.75 (10.13) | 1.78 (0.17) | |
| ***P*-value** | 0.591 | 0.112 | 0.780 | 0.728 | |
