# Supplementary figures and images for: Expression of miR-138 in cryopreserved bovine sperm is related to their fertility potential
Source: J Anim Sci Biotechnol. 2023 Sep 20;14:129. doi: 10.1186/s40104-023-00909-1 (PMC10510164; doi:10.1186/s40104-023-00909-1)

**Additional File 3.**

**A.**


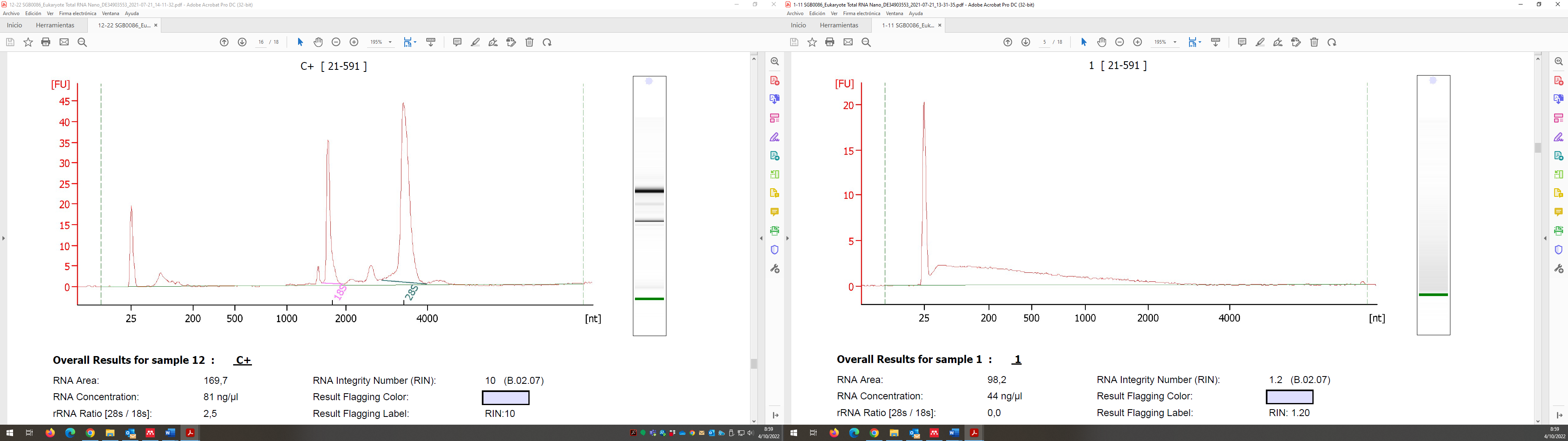

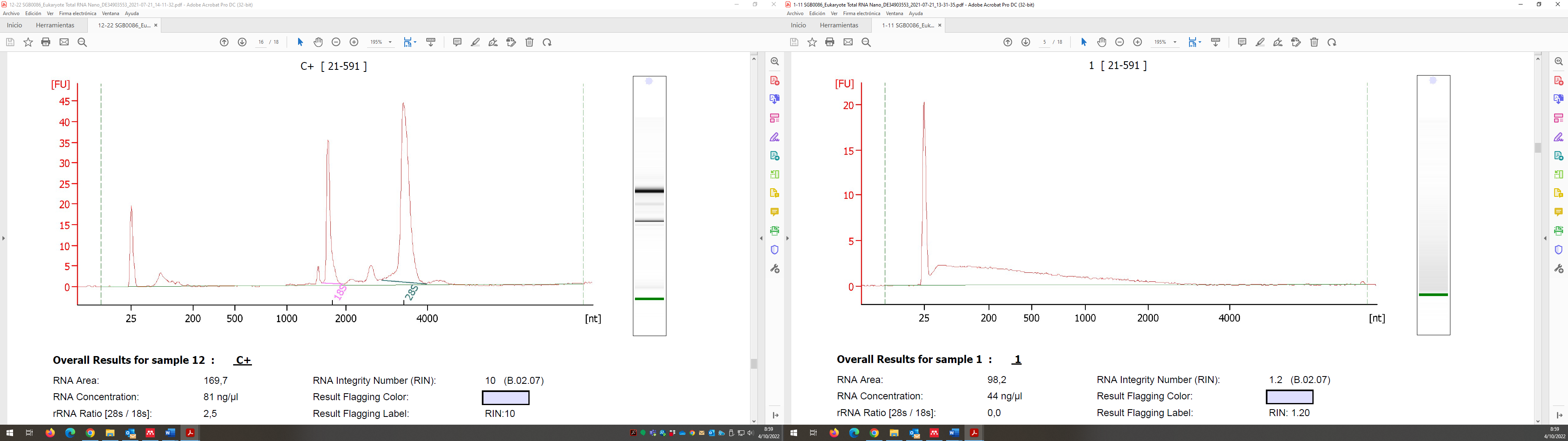


**B.**

Supplement: Supplementary file 3 — Additional file 3. Output of Bioanalyzer 2100 analysis. A Representative electropherogram of RNA isolated from Jurkatt cells and used as a positive control. B Representative electropherogram of RNA isolated form bovine sperm cells. Abbreviations: FU, fluorescence units; nt, nucleotides; RIN, RNA integrity number; rRNA, ribosomal RNA; S, svedberg sedimentation coefficient. [file 40104_2023_909_MOESM3_ESM.docx]

**Additional File 6.**


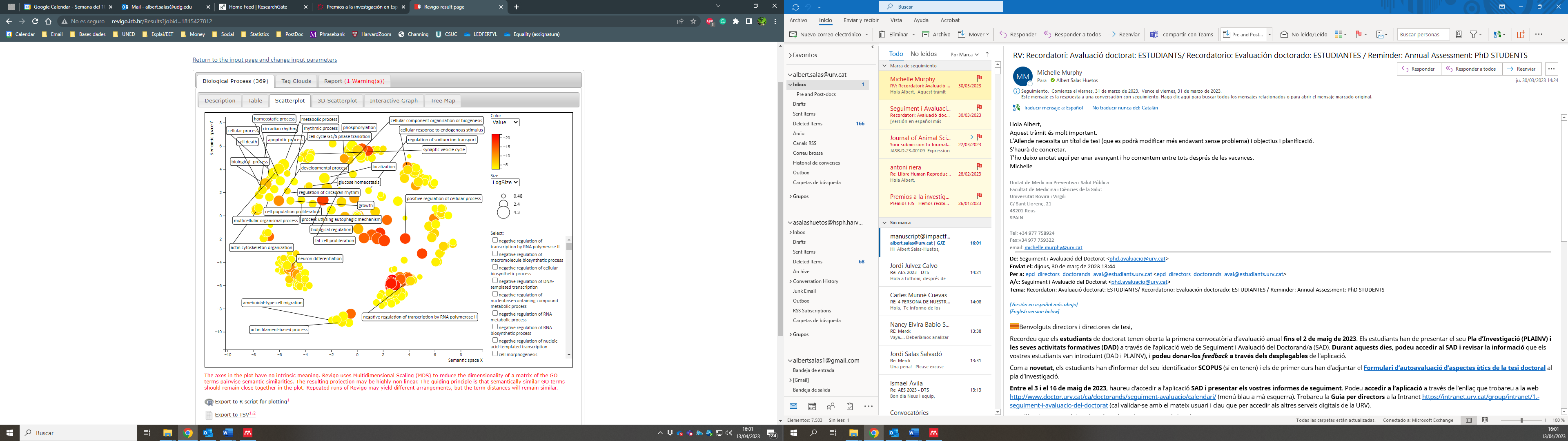

Supplement: Supplementary file 6 — Additional file 6. REVIGO clustering of Gene Ontology analysis of the predicted target genes for bta-miR-138. The axes in the plot have no intrinsic meaning. REVIGO uses Multidimensional Scalingto reduce the dimensionality of a matrix of the GO terms pairwise semantic similarities. [file 40104_2023_909_MOESM6_ESM.docx]
